# Advances and challenges of green hydrogen in Chile: a case study of the national strategy for sustainable energy transition

**DOI:** 10.14324/111.444/ucloe.3587

**Published:** 2026-08-06

**Authors:** Rodrigo Barra Novoa

**Affiliations:** 1 Andean-Lab, Valencia, Spain

**Keywords:** green hydrogen, energy transition, public policy, national strategy, renewable energy

## Abstract

Chile has emerged as one of the countries with the greatest potential for green hydrogen development due to its world-class renewable energy resources and the implementation of the National Green Hydrogen Strategy launched in 2020. The aim of this study is to analyse the coherence, achievements and limitations of this strategy as a public policy instrument designed to promote energy transition, decarbonisation and productive transformation. Drawing on a qualitative case study based on the systematic analysis of official documents, institutional reports and specialised literature, the research examines the institutional, regulatory, technological, economic and territorial dimensions shaping the development of the green hydrogen industry in Chile. The findings indicate that the country has established a relatively robust strategic and governance framework, accompanied by a rapid expansion of investment initiatives and increasing international visibility. Nevertheless, significant challenges remain, including cost competitiveness, technological dependency, transport and storage infrastructure, water availability, social acceptance of projects and uncertainty in international markets. The analysis demonstrates that the availability of renewable resources alone does not guarantee leadership in the hydrogen economy; institutional capacity, technological innovation, public–private coordination and territorial governance are equally important determinants of long-term success. The Chilean case provides valuable insights into the opportunities and constraints faced by emerging economies seeking to use green hydrogen as a tool for decarbonisation and sustainable industrial development.

## Introduction

Climate change constitutes one of the most pressing challenges of the 21st century. The accumulation of greenhouse gases (GHG) in the atmosphere, resulting from the combustion of fossil fuels, deforestation and carbon-intensive industrial practices, has caused unprecedented global warming. The Intergovernmental Panel on Climate Change (IPCC) warns that limiting global temperature rise to 1.5 °C above pre-industrial levels will require profound transformations in energy, transport and industrial systems [1,2].

In this context, green hydrogen (GH_2_) has emerged as one of the most promising technological pathways for the transition to a low-carbon economy. Unlike grey hydrogen, which is produced from fossil fuels without emissions mitigation, green hydrogen is generated through the electrolysis of water powered by renewable energy sources and produces no direct carbon dioxide (CO_2_) emissions. By contrast, blue hydrogen is also produced from fossil fuels – typically natural gas – but incorporates carbon capture and storage technologies to capture a significant proportion of the CO_2_ generated during production, thereby reducing its net greenhouse gas emissions [3].

While blue hydrogen also aims to reduce emissions by capturing and storing a significant share of the CO_2_ generated during production, its overall climate performance remains contingent on capture efficiency, upstream methane leakage and the long-term integrity of storage systems. Consequently, GH_2_ is widely regarded as one of the most robust long-term options for deep decarbonisation in sectors that are difficult to electrify directly.

Recent years have witnessed a rapid expansion of global interest in hydrogen as part of long-term decarbonisation strategies [4]. According to the International Energy Agency (IEA), global hydrogen demand has reached approximately 100 million tonnes per year, although the majority of production still relies on fossil fuels. Governments and industries across Europe, Asia and the Americas have announced national hydrogen strategies aimed at accelerating the deployment of low-carbon hydrogen technologies [5,6]. Hydrogen is expected to play a particularly important role in sectors that are difficult to electrify directly, including steel production, chemical manufacturing, long-distance transport, maritime shipping and aviation.

At the same time, the global hydrogen sector remains characterised by technological uncertainty, high capital costs and infrastructure constraints. While early policy discussions anticipated rapid market expansion, recent developments indicate that the deployment of large-scale hydrogen projects depends heavily on regulatory support, international cooperation, infrastructure availability and sustained investment. These challenges have led several governments and private actors to reassess project timelines and investment expectations, highlighting the complexity of building a globally competitive hydrogen economy.

Chile has gained increasing attention in the global race to develop GH_2_. Its unique combination of abundant solar and wind resources, macroeconomic stability and open trade policy has positioned the country as a potentially competitive producer within the emerging hydrogen economy. According to the Ministry of Energy (2022), Chile possesses a renewable energy potential exceeding 2 terawatts (TW) of installed capacity. In generation terms, this potential corresponds to several thousand terawatt-hours (TWh) per year, far exceeding domestic electricity demand and providing a substantial energy base for large-scale GH_2_ production [1].

The launch of the National Green Hydrogen Strategy (NGHS) in November 2020 marked a milestone in Chile’s energy policy. The strategy seeks to position the country among the world’s leading exporters of GH_2_ by 2040 while simultaneously reducing domestic emissions and fostering sustainable territorial development. Key targets included achieving production costs below USD 1/kg by 2030 and reaching 5 GW of electrolysis capacity under development by 2025 [1]. While these objectives reflected the optimism surrounding early hydrogen deployment scenarios, more recent assessments suggest that cost reductions may occur at a slower pace than initially anticipated.

By 2024, announced and pre-feasibility projects represented several gigawatts of planned electrolysis capacity, although only a fraction had reached advanced development stages. While the total pipeline of proposed projects suggests that the 5 GW target could be approached in the medium term, the current level of projects under construction remains more limited. This gap highlights both the ambition of the initial target and the structural challenges associated with permitting processes, infrastructure deployment and market formation in an emerging sector [1].

However, several structural challenges persist. Economically, the competitiveness of GH_2_ depends not only on reducing production costs – currently estimated between USD 3.0 and 7.5/kg, compared with USD 0.9 and 3.2/kg for grey hydrogen [7] – but also on addressing infrastructure constraints related to transport and storage. For countries seeking to export hydrogen over long distances, the logistics of maritime transport, conversion into hydrogen derivatives such as ammonia, and associated energy losses may represent significant bottlenecks. In addition, the sector remains highly dependent on imported electrolysers and specialised equipment, limiting domestic value capture and exposing projects to global supply-chain disruptions. Institutionally, the development of a clear regulatory framework and effective governance mechanisms remains essential. Socially and environmentally, community acceptance, sustainable water management and ecological safeguards must also be ensured.

This case study examines the implementation of Chile’s NGHS from a public-policy perspective, assessing its institutional coherence, governance arrangements, technological aspirations and emerging implementation challenges within the broader context of sustainable energy transition. Despite the growing literature on hydrogen economics, techno-economic feasibility and national hydrogen roadmaps, relatively few studies have examined how developing countries translate ambitious hydrogen strategies into institutional arrangements, territorial governance mechanisms and industrial development pathways. Recent reviews of the Chilean hydrogen sector have focused primarily on deployment trends, cost competitiveness and market integration, while less attention has been devoted to analysing the NGHS as a comprehensive public-policy framework connecting decarbonisation, industrial transformation and territorial development. This study addresses this gap by providing an integrated policy-oriented assessment of the Chilean experience, examining the interaction between governance structures, technological development, economic opportunities and implementation constraints that may shape the long-term success of the hydrogen transition [8].

**Figure 1:**
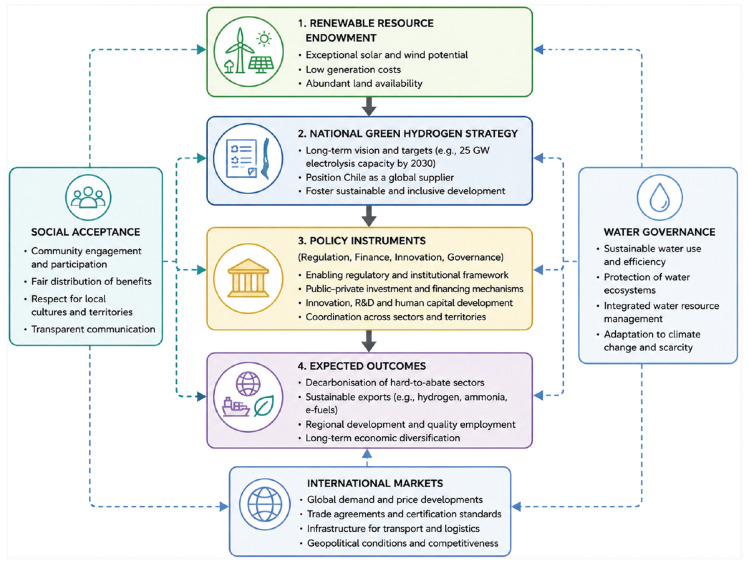
Conceptual framework of Chile’s GH_2_ transition. Source: author's own elaboration based on the National Green Hydrogen Strategy [1], recent literature on green hydrogen development [8,9], and the qualitative analysis conducted in this study.

To guide the analysis, Fig. 1 presents the conceptual framework adopted in this study. The framework illustrates how Chile’s renewable-resource endowment is translated into strategic policy objectives through the NGHS and its associated policy instruments, while highlighting the moderating influence of social acceptance, water governance and international market conditions on the achievement of expected outcomes.

## Materials and methods

This study adopts a qualitative case study approach to examine the development of GH_2_ policy in Chile. The analysis focuses on the NGHS as the principal public-policy framework guiding the country’s transition towards a low-carbon energy system. The objective is not to produce original quantitative measurements but to assess the institutional design, strategic objectives and implementation challenges associated with this policy initiative.

The research is based on a systematic documentary analysis of official policy documents, institutional reports, technical studies and academic literature related to hydrogen development and energy transition. The primary source is the report *Green Hydrogen – A National Project* published by the Chilean Ministry of Energy in 2022 [1], which was selected because it represents the official roadmap for hydrogen deployment in Chile.

To strengthen the analytical basis of the study, this core document was complemented with publications and datasets from national and international institutions, including the National Energy Commission, the Production Development Corporation (CORFO), the German Agency for International Cooperation (GIZ) [10], the International Energy Agency and the Inter-American Development Bank [11]. The documentary corpus consisted of more than 30 sources published between 2020 and 2025.

Source selection followed three criteria: (a) direct relevance to the formulation or implementation of the NDHS; (b) publication by recognised governmental, international or academic institutions; and (c) contribution to the analysis of regulatory, economic, technological or governance dimensions of hydrogen development.

The analytical procedure was conducted in four stages. First, the selected documents were systematically reviewed to identify the main policy objectives, institutional actors, regulatory instruments and implementation mechanisms associated with the NGHS.

Second, the information collected was organised through an analytical categorisation process structured around three dimensions: (a) institutional and regulatory developments; (b) economic and technological dynamics; and (c) social, environmental and governance challenges.

Third, a triangulation process was undertaken by comparing national policy documents with international reports and peer-reviewed academic literature. This procedure allowed the identification of convergences and divergences across sources, reducing the risk of institutional bias and improving the robustness of the analysis.

Fourth, an interpretative policy-analysis framework was applied using concepts from public-policy studies [12]. This stage examined the alignment between policy objectives, implementation instruments and observable developments in the hydrogen sector, enabling an assessment of the coherence, opportunities and limitations of the Chilean strategy.

Given the qualitative nature of the research, the findings should be interpreted as policy-oriented insights rather than statistical generalisations. The purpose of the study is to evaluate the strategic consistency and implementation challenges of Chile’s hydrogen policy within the broader context of sustainable energy transition.

## Results

### Global context of GH_2_

The development of GH_2_ has gained significant momentum following the global commitments under the 2015 Paris Agreement. Countries such as Germany, Japan, Australia and South Korea have identified hydrogen as a key enabler for carbon neutrality, particularly in energy-intensive industries [13].

At present, hydrogen produced via renewable electrolysis accounts for less than 0.1% of global hydrogen output. Nevertheless, projections have been highly optimistic: the Hydrogen Council estimates that the worldwide hydrogen market could generate annual revenues of USD 2.5 trillion and create more than 30 million jobs by 2050 [14].

However, recent developments suggest a more complex and uncertain trajectory for the hydrogen economy. In the past two years, several large-scale projects have faced delays, cancellations or revisions due to rising capital costs, slower-than-expected demand growth and uncertainties surrounding infrastructure deployment and regulatory frameworks. These developments indicate that the expansion of the hydrogen sector will likely occur more gradually than initially anticipated.

Chile participates in this global dynamic from a position of significant comparative advantage. Its electricity matrix already contains over 60% renewable generation, and its geographical diversity provides unique conditions for low-cost renewable energy. The Atacama Desert offers the world’s highest solar irradiance, while the Magallanes Region features strong and consistent winds exceeding 60% capacity factors, comparable to leading offshore sites in Europe [1,14].

At the same time, Chile also faces structural challenges that may influence its competitiveness in global hydrogen markets. One of the most significant constraints is its geographical distance from major demand centres in Europe and Asia, which implies reliance on long-distance maritime transport of hydrogen or hydrogen derivatives. These logistical requirements may increase costs and introduce additional energy losses, potentially affecting the economic viability of large-scale hydrogen exports.

### Renewable potential and comparative advantages

Chile’s total renewable energy potential surpasses 1800 GW, equivalent to approximately 80 times its current electricity demand [1]. This endowment provides the country with favourable conditions for large-scale GH_2_ production. Early studies suggested that Chile could become one of the lowest-cost producers of GH_2_ globally. For instance, a study by McKinsey & Company (2020) estimated that production costs could fall below USD 1/kg by 2030 under favourable technological and market conditions [15].

More recent analyses, however, indicate that cost projections remain uncertain and may be higher than initially anticipated due to rising capital costs, infrastructure requirements and market uncertainties. Consequently, many recent studies suggest that production costs closer to USD 1.5–2.5/kg by 2030 may represent a more realistic range depending on technological progress and policy support.

### Institutional and regulatory advances

The institutional architecture underpinning Chile’s hydrogen development is robust and evolving. The Ministry of Energy leads implementation through the Division of Sustainable Energy and coordinates the Interministerial Committee on Green Hydrogen, integrating the Ministries of Finance, Economy, Science and Environment [1].

The NGHS defines three progressive stages:

1. 2020–2025: pilot projects, regulatory design and initial capacity building.
2. 2025–2030: commercial expansion, infrastructure development and integration into global markets.
3. 2030–2050: consolidation as a global exporter and domestic industry maturity [1].

Regulatory progress includes:

- The Framework Law on Climate Change (2022), enshrining carbon neutrality by 2050.
- The Energy Flexibility Strategy (2021), promoting renewable integration.
- The National Electromobility Policy (2022), mandating that all light and medium vehicle sales be zero-emission by 2035 [16].

CORFO has been central to this institutional build-up. In 2021 it launched the first public tender for CH_2_ projects, allocating USD 50 million in co-financing for six pioneering projects totalling over 400 MW of electrolysis capacity, representing around USD 1 billion in total investment [1].

Moreover, the Chilean Hydrogen Council (H2 Chile), established in 2022, serves as a multi-stakeholder governance platform bringing together government, industry, academia and civil society.

Chile’s international partnerships amplify its institutional reach. Bilateral cooperation with Germany, the European Union, Japan and Norway has facilitated technical assistance, sustainability certification frameworks and access to green finance [5]. GIZ and the KfW Development Bank have supported feasibility studies and capacity-building programmes across key regions.

### Flagship projects and international partnerships

Recent assessments indicate that more than 80 hydrogen-related projects have been announced nationwide. However, only a limited number have progressed through environmental approval processes or reached advanced implementation stages. This distinction between announced projects and operational deployment is important because it highlights the gap between strategic ambitions and actual market development [8].

Notable initiatives include:

- Haru Oni (Magallanes): led by Highly Innovative Fuels (HIF) Global, Siemens Energy, Enel Green Power and Porsche, this project integrates renewable generation, carbon capture and e-fuel production. Its pilot phase (2022) achieved 130,000 litres of synthetic fuel output, with a commercial expansion planned to reach 550 million litres by 2027 [17].
- HyEx (Antofagasta): developed by Engie and ENAEX, aims to replace diesel in ammonium-nitrate production for copper mining. The project’s initial 30 MW phase could scale to 800 MW [18].
- Cabo Negro (Magallanes): a partnership between ENAP and Total Eren for ammonia production with a projected capacity of 500 MW [19].
- HIF Atacama: producing green methanol and synthetic hydrocarbons for export markets.

These projects signal Chile’s transition from pilot testing to early commercialisation. The strong presence of foreign capital and technology partners, while beneficial for scale, also raises concerns regarding domestic value capture and technological dependency [20].

In response, public policy increasingly promotes local supply-chain participation and public–private collaboration, aiming to transform foreign investment into long-term domestic capabilities rather than enclave operations.

### Economic impact and projections

The development of GH_2_ has the potential to transform Chile’s productive structure and its integration into the global energy economy. According to projections by the Ministry of Energy [1], the domestic hydrogen sector could generate over 100,000 direct and indirect jobs by 2030, and export revenues exceeding USD 24 billion annually by 2050 – equivalent to nearly 10 per cent of the country’s current GDP.

The sector’s multiplier effect extends across a diverse set of industries:

- Manufacturing: production of electrolysers, valves, storage systems and associated electrical components.
- Engineering and construction: design and maintenance of hydrogen plants and infrastructure.
- Logistics: port and transport systems for ammonia, methanol and liquid hydrogen.
- Energy derivatives: development of synthetic fuels and fertilisers based on GH_2_.

Regionally, GH_2_ could become a driver of diversification for territories historically dependent on extractive activities. Northern regions such as Antofagasta and southern Magallanes – blessed with exceptional solar and wind resources, respectively – are expected to host the largest clusters of production. This could foster new regional development poles, enhance skilled employment and reduce economic asymmetries [13].

However, several economic challenges remain. The levelised cost of hydrogen continues to be relatively high, ranging between USD 3 and 5/kg H_2_ under current technological and market conditions, compared with grey hydrogen at approximately USD 1–2/kg H_2_. These figures differ from the lower cost projections discussed earlier in the paper, which refer to potential cost reductions by 2030 under favourable technological learning and large-scale deployment scenarios. Significant capital investment and continued technological learning will therefore be required to close this gap [9]. Moreover, the cost of water desalination and compression, as well as the absence of export pipelines and dedicated port infrastructure, add further complexity to scaling up hydrogen production and export.

The Chilean policy framework seeks to mitigate these barriers through a combination of public co-financing (via CORFO) and risk-sharing mechanisms that attract foreign direct investment while nurturing domestic capacity. Yet, achieving long-term competitiveness will depend on whether Chile can evolve from a resource-based exporter to a knowledge-driven innovator within the hydrogen value chain.

### Summary of key findings

Table 1 synthesises the principal findings of this study regarding the development of GH_2_ in Chile, classified across five analytical dimensions: institutional, economic, technological, social–environmental and international.

**Table 1.** Summary of key findings on the development of the GH_2_ sector in Chile

Dimension	Key findings and evidence	Implications for Chile’s GH_2_ strategy
Institutional and regulatory framework	The NGHS [1] and the National Hydrogen Council have established a long-term policy framework for sector development. Regulatory initiatives related to safety standards, permitting coordination and carbon-pricing discussions have contributed to reducing uncertainty for investors and improving institutional coordination.	A stable regulatory environment supports investor confidence; however, continued progress will require stronger interministerial coordination, regional implementation capacity and regulatory adaptation as projects move from planning to deployment.
Economic and industrial development	More than 80 hydrogen-related projects had been announced by 2024, although only a limited number had reached advanced development stages. Government scenarios project investments that could exceed USD 45 billion under favourable deployment conditions. Early support programmes, including CORFO co-financing initiatives, contributed to the development of pilot projects and the emergence of the domestic hydrogen ecosystem [21]. Recent assessments indicate that future hydrogen production costs will depend on technological learning, infrastructure availability, financing conditions and international market developments, with cost projections remaining subject to significant uncertainty [8,9].	The sector presents opportunities for industrial diversification, export development and regional economic growth. However, realising these benefits will require strengthening domestic technological capabilities, developing local supply chains, fostering innovation ecosystems and expanding specialised human capital to maximise local value creation and reduce technological dependence.
Technological progress	Pilot and demonstration projects such as Haru Oni (synthetic fuels), HyEx (green ammonia) and Cabo Negro (ammonia export) illustrate progress in technology deployment and industrial experimentation within Chile’s emerging hydrogen sector. However, the industry continues to rely heavily on imported electrolysers and specialised equipment, limiting domestic technological participation and value capture [8,22].	Strengthening domestic research and development capabilities, promoting technology transfer and fostering collaboration between universities, industry and government will be critical to enhancing local innovation capacity, reducing technological dependence and supporting the long-term competitiveness of the hydrogen sector.
Social, environmental and territorial governance	Stakeholders in regions such as Magallanes and Antofagasta increasingly demand transparency, participation and equitable benefit-sharing mechanisms. Water-resource management has emerged as a key challenge, particularly in arid regions where hydrogen production may compete with other economic activities. Desalination and circular-economy approaches are being considered to address these constraints [8,18].	Long-term project viability will depend on socially legitimate governance arrangements, effective stakeholder engagement and integrated territorial planning capable of balancing economic development with environmental sustainability.
International positioning and cooperation	Strategic partnerships with Germany, the European Union, Japan and South Korea have supported certification initiatives, technology cooperation and market-development activities. International collaboration has strengthened Chile’s visibility within emerging hydrogen value chains and facilitated access to knowledge and investment networks [1].	International engagement may enhance market access and competitiveness, but future export opportunities will depend on compliance with evolving sustainability standards, certification requirements and global market conditions.

Overall, the evidence suggests that Chile has established a robust policy framework for GH_2_ development, supported by favourable renewable-resource endowments and increasing international cooperation. Nevertheless, technological dependence, infrastructure constraints, territorial governance and innovation capacity remain critical challenges that may influence the long-term effectiveness of the NGHS.

### Structural barriers and policy responses

Beyond its evident opportunities, the development of a GH_2_ industry in Chile faces several structural barriers that may influence the long-term viability of the sector. Among the most significant challenges are the high upfront capital costs associated with large-scale electrolysis projects, the current dependence on imported technological components such as electrolysers, and the need for substantial investment in transport, storage and export infrastructure. In addition, the geographical distance between Chile and major demand centres in Europe and Asia increases logistical complexity and transport costs, particularly for hydrogen derivatives shipped over long maritime routes.

Transport and export logistics constitute another major constraint. The geographical distance separating Chile from key demand centres in Europe and Asia implies additional costs associated with storage, conversion into hydrogen carriers such as ammonia or methanol, maritime transport and reconversion processes. These factors may significantly affect final delivered costs and therefore represent a critical determinant of international competitiveness [8].

A further challenge concerns technological dependency. Although Chile possesses exceptional renewable resources, most electrolysers, specialised components and engineering systems continue to be imported. This dependence may limit domestic value capture and expose projects to international supply-chain disruptions, underscoring the importance of fostering local manufacturing capabilities, technological learning and applied research ecosystems [8].

Institutional and social dimensions also represent critical factors. The expansion of hydrogen projects in regions such as Antofagasta and Magallanes requires effective mechanisms of territorial governance, environmental management and community participation. Issues related to water use, land occupation and the distribution of economic benefits have become central topics in local debates. Addressing these barriers will require coordinated policy responses, including targeted public financing mechanisms, investment in infrastructure, support for domestic technological capabilities and participatory governance frameworks that enhance social legitimacy [23].

## Discussion

### Technological and economic challenges

Chile’s GH_2_ pathway is underpinned by an explicit ‘mission-oriented policy’ framework [24], in which the state acts not as a market substitute but as an enabler and coordinator of a multi-sectoral industrial transition. This mission-driven orientation corresponds to the first of the six policy pillars in the NGHS, highlighting the government’s catalytic role in reducing regulatory, financial and technical uncertainty.

From a technological perspective, progress in electrolysis efficiency and cost reduction remains essential. Early projections suggested significant declines in electrolyser capital costs as manufacturing scales up. For instance, a study by McKinsey Company for the Chilean Ministry of Energy estimated that electrolyser capital costs could fall by up to 70% by 2030 under favourable industrial scaling and automation scenarios [9].

More recent analyses, however, indicate that the pace of cost reduction remains uncertain due to fluctuations in supply chains, financing costs and global demand for critical materials. As a result, while technological learning continues to drive improvements in electrolyser performance, achieving the most optimistic cost reduction targets will depend on sustained investment, industrial deployment and supportive policy frameworks.

Achieving these objectives will require not only external investment but also the development of domestic manufacturing capabilities – a focus explicitly incorporated in Chile’s industrial pillar aimed at generating local value through innovation and technology transfer.

Economically, the country must continue to strengthen its green-finance architecture. The NGHS action plan proposes the establishment of public–private financing mechanisms, including blended-finance models, concessional credit lines and green-bond issuance, to de-risk early-stage projects and bridge cost gaps with fossil-fuel alternatives [1]. These instruments align with the ‘clean-export-economy’ pillar, designed to ensure that Chile’s renewable advantage translates into globally competitive, low-carbon products.

However, the volatility of global markets still affects investor confidence. To sustain competitiveness, Chile will need to diversify its portfolio of projects and pursue vertical integration – from renewable generation to electrolyser manufacturing and derivative-fuel production – thus capturing a greater share of the emerging hydrogen value chain.

### Governance, social acceptance and sustainability

The NGHS explicitly recognises that technological leadership must coexist with territorial balance and environmental stewardship. The pillar of ‘balanced use of resources and territory’ emphasises the need to align industrial expansion with ecological protection and sustainable resource management [1]. In practice, this requires hydrogen projects to incorporate early environmental assessments, transparent consultation processes and adaptive governance mechanisms capable of addressing local concerns.

Water availability represents one of the most significant sustainability challenges for large-scale hydrogen deployment. Electrolysis requires approximately 9L of water per kilogram of hydrogen produced, and many of the regions with the highest renewable-energy potential are characterised by water stress. Consequently, future projects are expected to rely heavily on seawater desalination, recycling systems and integrated water-management strategies. While desalination provides a technically feasible solution, it also increases capital and operating costs and introduces additional environmental and territorial considerations that require careful planning and governance [8].

Social acceptance has emerged as an equally important condition for the long-term viability of the sector. The NGHS identifies hydrogen development as a potential driver of regional development through the creation of innovation ecosystems, local supplier networks and specialised employment opportunities. However, recent evidence suggests that social legitimacy cannot be assumed and must be actively built through transparent decision-making processes, meaningful stakeholder participation and equitable benefit-sharing arrangements. In regions such as Antofagasta and Magallanes, communities increasingly demand a greater role in discussions concerning land use, environmental impacts and the distribution of economic benefits associated with hydrogen projects [8].

To address these challenges, Chile has established institutional mechanisms such as the National Green Hydrogen Council, a public–private platform responsible for coordinating implementation, monitoring progress and facilitating dialogue among stakeholders. Nevertheless, achieving socially legitimate and territorially balanced development will require stronger decentralised governance capacities and more effective mechanisms for community engagement and benefit sharing [17].

### Human capital, innovation and institutional learning

The fifth pillar of the NGHS – capacity building and innovation – acknowledges that the hydrogen transition is as much a knowledge challenge as a technological one. The strategy’s action plan proposes three complementary measures: (a) connecting universities, industry and vocational institutions to identify skill gaps; (b) developing a national RD roadmap for hydrogen technologies; and (c) creating training programmes for public-sector officials in regulation, inspection and safety [1].

Progress has begun through collaboration with GIZ and the Clean Technologies Institute, which is financed with up to USD 193 millions of public funds [10]. Nevertheless, coordination between higher-education providers and industrial demand remains partial. Establishing a National Hydrogen Technology Centre, as planned by the Ministry of Energy, would institutionalise learning processes and position Chile as a knowledge exporter within Latin America [25].

Beyond technical education, the strategy underscores innovation as a cross-cutting instrument of competitiveness. By fostering start-ups and supporting applied research, Chile could shift from a model of resource extraction to one of technological production, capturing intellectual-property value in emerging hydrogen applications [26].

### International positioning and ‘co-opetition’

The final pillar of the NGHS, international openness, embodies Chile’s long-standing economic diplomacy. The strategy calls for a proactive ‘hydrogen diplomacy’, leveraging Chile’s 60 trade agreements and 171 diplomatic relationships to attract investment, share technology and negotiate export markets [1].

This ‘co-opetition’ paradigm – cooperation with potential competitors – aims to accelerate the scaling of the global hydrogen economy while establishing Chile as a trusted supplier of certified low-carbon fuels. The government has already signed memoranda of understanding with Germany, the European Union, Japan and South Korea to collaborate on research, certification and logistics.

International cooperation thus reinforces domestic objectives: the development of a new clean-export economy that adds ‘green value’ to Chilean products such as copper, fertilisers and synthetic fuels. Through transparent standards and sustainability certification, Chile can differentiate its exports and strengthen its brand as a renewable-energy nation [27].

This international positioning also reflects broader global trends in which hydrogen is increasingly recognised as a strategic component of future low-carbon energy systems, although countries are expected to follow different technological and commercial pathways depending on their comparative advantages [28].

## Conclusions

Chile’s experience with GH_2_ illustrates how an emerging economy can mobilise renewable-energy resources, public policy and international cooperation to pursue a low-carbon development strategy. Since the launch of the NGHS in 2020, the country has established an ambitious policy framework aimed at linking decarbonisation objectives with industrial development, export diversification and territorial sustainability. The six strategic pillars of the NGHS provide a coherent structure for coordinating regulatory, technological, economic and governance initiatives within a long-term energy transition agenda [1].

The analysis shows that Chile has made significant progress in creating the institutional conditions required for the development of a GH_2_ industry. Multi-ministerial coordination, public–private collaboration mechanisms and international partnerships have strengthened the visibility of the sector and attracted substantial investor interest. More than 80 hydrogen-related projects have been announced nationwide, and government scenarios project investments that could exceed USD 45 billion under favourable deployment conditions. However, only a limited number of projects have reached advanced implementation stages, highlighting the gap that still exists between strategic ambitions and realised investments [8].

At the same time, the findings reveal that the long-term success of the hydrogen sector will depend on the effective management of several structural challenges. These include the competitiveness of production costs, the development of transport and export infrastructure, dependence on imported technologies, water-resource management and the social legitimacy of large-scale projects. While Chile benefits from exceptional solar and wind resources, renewable-energy potential alone is insufficient to guarantee international leadership. Institutional capacity, technological innovation, public–private coordination and territorial governance emerge as equally important determinants of long-term competitiveness [8].

The study also highlights the importance of integrating economic, environmental and social dimensions within hydrogen policy design. In regions such as Antofagasta and Magallanes, the sustainability of future projects will depend on transparent governance mechanisms, meaningful stakeholder participation and equitable benefit-sharing arrangements. Similarly, strengthening domestic research and development capabilities, fostering technological learning and promoting local value creation will be essential to maximise the developmental benefits associated with hydrogen deployment.

Overall, the Chilean case illustrates both the opportunities and the limitations of hydrogen-based development strategies in resource-rich emerging economies [4]. While the NGHS has established a robust strategic framework, its long-term effectiveness will depend on the country’s ability to translate policy objectives into implemented projects, technological capabilities and socially legitimate development outcomes. Future research should focus on project-level implementation, infrastructure deployment, social acceptance dynamics and the evolution of international hydrogen markets in order to assess whether current policy expectations translate into sustained economic, environmental and territorial benefits.

## Data Availability

The datasets generated during and/or analysed during the current study are available from the corresponding author on reasonable request. The core materials analysed – including the Estrategia Nacional de Hidrógeno Verde (2020) and Green Hydrogen – A National Project (2022) – are freely available on the official website of the Chilean Ministry of Energy (https://energia.gob.cl).

## References

[1] Ministerio de Energía de Chile (2020). National green hydrogen strategy [online].

[2] Intergovernmental Panel on Climate Change (IPCC) (2023). Climate change 2021 – the physical science basis [online].

[3] International Energy Agency (IEA) (2022). Global hydrogen review 2022 [online].

[4] Economic Commission for Latin America and the Caribbean (ECLAC); H2Lac (2023). Renewable hydrogen in Latin America and the Caribbean: opportunities, challenges and pathways [online].

[5] European Commission (2020). A hydrogen strategy for a climate neutral Europe [online].

[6] Department of Climate Change, Energy, the Environment and Water (Australia) (2019). Australia’s National Hydrogen Strategy [online].

[7] BloombergNEF (2023). Hydrogen economy outlook: key messages [online].

[8] Schneider H, Chamy R, Valderrama C, Morales A, Farías F, Vinardell S (2026). Green hydrogen development in Chile: a review of deployment, techno-economics, and global market integration. Clean Technol [online].

[9] McKinsey Company (2020). Chilean hydrogen pathway.

[10] Deutsche Gesellschaft für Internationale Zusammenarbeit (GIZ) (2023). Programa de energías renovables y eficiencia energética – 4e en Chile: informe de cooperación 2018–2023 [online].

[11] Inter-American Development Bank (IDB) (2023). Unlocking green and just hydrogen in Latin America and the Caribbean [online].

[12] Howlett M, Ramesh M, Perl A (2020). Studying public policy: policy cycles and policy subsystems.

[13] Comisión Nacional de Energía (CNE) (2023). Balance nacional de energía 2023 [online].

[14] Hydrogen Council (2017). Hydrogen scaling up: a sustainable pathway for the global energy transition [online].

[15] International Renewable Energy Agency (IRENA) (2020). Green hydrogen cost reduction: scaling up electrolysers to meet the 1.5°C climate goal [online].

[16] Organisation for Economic Co-operation and Development (OECD) (2019). Financing climate futures: rethinking infrastructure [online].

[17] Sovacool BK, Griffiths S (2020). Culture and low-carbon energy transitions. Nat Sustain [online].

[18] World Bank (2022). Advancing knowledge of the water-energy nexus in the GCC countries [online].

[19] Ministerio de Educación de Chile (2023). Informe sobre Formación Técnica y Capital Humano en Energías Renovables.

[20] Universidad de Magallanes (2023). Brechas programáticas y de infraestructura para la capacitación y formación de capacidades humanas para la industria de hidrógeno verde en la Región de Magallanes y la Antártica Chilena [online].

[21] Corporación de Fomento de la Producción (CORFO) (2021). First call for financing of green hydrogen projects in Chile [online].

[22] Enel Green Power, Siemens Energy (2023). Haru Oni project report: synthetic fuels in Magallanes.

[23] Energy Transitions Commission (2021). Making the hydrogen economy possible [online].

[24] Mazzucato Mariana (2021). Mission economy: a moonshot guide to changing capitalism.

[25] Ministerio de Energía de Chile (2024). Propuesta de Creación del Centro Nacional de Tecnologías del Hidrógeno.

[26] United Nations Industrial Development Organization (UNIDO) (2023). Global programme for hydrogen in industry [online].

[27] International Partnership for Hydrogen and Fuel Cells in the Economy (IPHE) (2024). Hydrogen certification 101 [online].

[28] World Energy Council (WEC) (2022). World energy issues monitor 2022 [online].

